# Molecular Diagnosis of *Brettanomyces bruxellensis*’ Sulfur Dioxide Sensitivity Through Genotype Specific Method

**DOI:** 10.3389/fmicb.2018.01260

**Published:** 2018-06-11

**Authors:** Marta Avramova, Amélie Vallet-Courbin, Julie Maupeu, Isabelle Masneuf-Pomarède, Warren Albertin

**Affiliations:** ^1^USC 1366 INRA, Institut des Sciences de la Vigne et du Vin, Unité de Recherche Œnologie EA 4577, University of Bordeaux, Bordeaux, France; ^2^School of Agriculture, Food and Wine, The University of Adelaide, Adelaide, SA, Australia; ^3^Microflora-ADERA, Institut des Sciences de la Vigne et du Vin, Unité de Rrecherche Œnologie EA 4577, Bordeaux, France; ^4^Bordeaux Sciences Agro, Gradignan, France; ^5^École Nationale Supérieure de Chimie de Biologie et de Physique, Institut Polytechnique de Bordeaux, Bordeaux, France

**Keywords:** *Brettanomyces bruxellensis*, resistance, tolerance, sulfur dioxide, wine, spoilage yeast

## Abstract

The yeast species *Brettanomyces bruxellensis* is associated with important economic losses due to red wine spoilage. The most common method to prevent and/or control *B. bruxellensis* spoilage in winemaking is the addition of sulfur dioxide into must and wine. However, recently, it was reported that some *B. bruxellensis* strains could be tolerant to commonly used doses of SO_2_. In this work, *B. bruxellensis* response to SO_2_ was assessed in order to explore the relationship between SO_2_ tolerance and genotype. We selected 145 isolates representative of the genetic diversity of the species, and from different fermentation niches (roughly 70% from grape wine fermentation environment, and 30% from beer, ethanol, tequila, kombucha, etc.). These isolates were grown in media harboring increasing sulfite concentrations, from 0 to 0.6 mg.L^-1^ of molecular SO_2_. Three behaviors were defined: sensitive strains showed longer lag phase and slower growth rate and/or lower maximum population size in presence of increasing concentrations of SO_2_. Tolerant strains displayed increased lag phase, but maximal growth rate and maximal population size remained unchanged. Finally, resistant strains showed no growth variation whatever the SO_2_ concentrations. 36% (52/145) of *B. bruxellensis* isolates were resistant or tolerant to sulfite, and up to 43% (46/107) when considering only wine isolates. Moreover, most of the resistant/tolerant strains belonged to two specific genetic groups, allowing the use of microsatellite genotyping to predict the risk of sulfur dioxide resistance/tolerance with high reliability (>90%). Such molecular diagnosis could help the winemakers to adjust antimicrobial techniques and efficient spoilage prevention with minimal intervention.

## Introduction

Winemakers manage the transformation of must into wine through various processes, aiming to obtain high quality product according to their wishes and the expectations of their customers. However, wine chemical and microbiological properties are in constant evolution throughout the winemaking process, and some parameters are difficult to control. Yeast metabolism is one of the multiple factors shaping wine aromatic and flavor properties by contributing to its complexity or, in some cases, leading to undesirable aromas (Fleet, 2003). One example of such phenomenon is wine spoilage by *Brettanomyces bruxellensis*, a yeast species related to production of off-aromas perceived as barnyard, horse sweat, or medicinal (Heresztyn, 1986; Chatonnet et al., 1992). Prevention methods against *B. bruxellensis* development include spoilage risk evaluation, SO_2_ addition, the use of biocontrol agents, e.g., through the inoculation/co-inoculation of various species and/or strains of yeast and bacteria (Berbegal et al., 2017, 2018), etc. If *B. bruxellensis* is detected, different elimination techniques exist which could be roughly divided in physical (filtering, the use of electric current, pressure, temperature, ultrasonics, etc.) and chemical (SO_2_, chitosan, DMDC, yeast-derived killer toxins, etc.), see for details (Delfini et al., 2002; Lustrato et al., 2010; Francesca and Maurizio, 2011; Luo et al., 2012; Umiker et al., 2013; Mehlomakulu et al., 2014; Fabrizio et al., 2015; Taillandier et al., 2015; González-Arenzana et al., 2016, 2018; Petrova et al., 2016; Berbegal et al., 2017). Still, the most common method to prevent and/or control *B. bruxellensis* spoilage remains the addition of sulfur dioxide into must and wine, with regular adjustments if needed. Sulfites are used in winemaking at least since the 18th century and are introduced either through the burning of sulfur tablets in barrels, or in liquid form, mainly through addition of potassium bisulfite solution to must and wine (Ribéreau-Gayon et al., 2006). Sulfur dioxide is broadly used in winemaking not only for its antiseptic action, but also for its antioxidant and antioxidasic properties (Ribéreau-Gayon et al., 2006). Thus, SO_2_ addition is the preferred choice when it comes to *B. bruxellensis* spoilage prevention. Unfortunately, over the last years, some *B. bruxellensis* strains were reported to be tolerant to commonly used doses of SO_2_, with a high variability amongst isolates (Barata et al., 2008; Curtin et al., 2012; Agnolucci et al., 2014). This variability makes the prediction of *B. bruxellensis* spoilage potential and the choice of adequate antimicrobial agent a challenge for winemakers. Recently, it was shown that *B. bruxellensis* SO_2_ sensitivity correlates with genotype defined by both AFLP and microsatellite markers (Curtin et al., 2012; Avramova et al., 2018). The former study analyzed a total of 41 isolates, with a focus on Australian wine strains. The latter study assessed the intraspecific genetic diversity of a larger number of isolates (1488 strains from 29 countries and 5 types of fermentation niches). Microsatellite genotype analysis revealed that the population was structured according to ploidy level (some clusters being mainly composed of diploid isolates, whereas others – of triploid ones). Statistical analysis of the generated data highlighted that both substrate of isolation and geographical origin of the isolates contribute to the observed population structure. The results suggested an anthropic influence on the spatial biodiversity of *B. bruxellensis*. The hypothesis of human-related factors effect on the population was further supported by the correlation between genotypic clustering and tolerance to SO_2_, the main antimicrobial agent used by winemakers. In particular, among the six main clusters of *B. bruxellensis* population (Avramova et al., 2018), two genetic clusters (AWRI1499-like and L0308-like) were highlighted to comprise isolates with high SO_2_ tolerance (Avramova et al., 2018). However, SO_2_ sensitivity was tested on a limited number of isolates (39), particularly for the L0308-like cluster (2 isolates). Thus, the aims of this study were (i) to extend the screening of SO_2_ sensitivity to 106 additional isolates and thus confirm/infirm the correlation between genetic clusters and SO_2_ sensitivity to a larger collection representative of the global *B. bruxellensis* population and (ii) to validate the applicability of a method allowing the prediction of *B. bruxellensis* SO_2_ sensitivity through genetic markers analysis.

## Materials and Methods

### Strains

**FIGURE 1:**
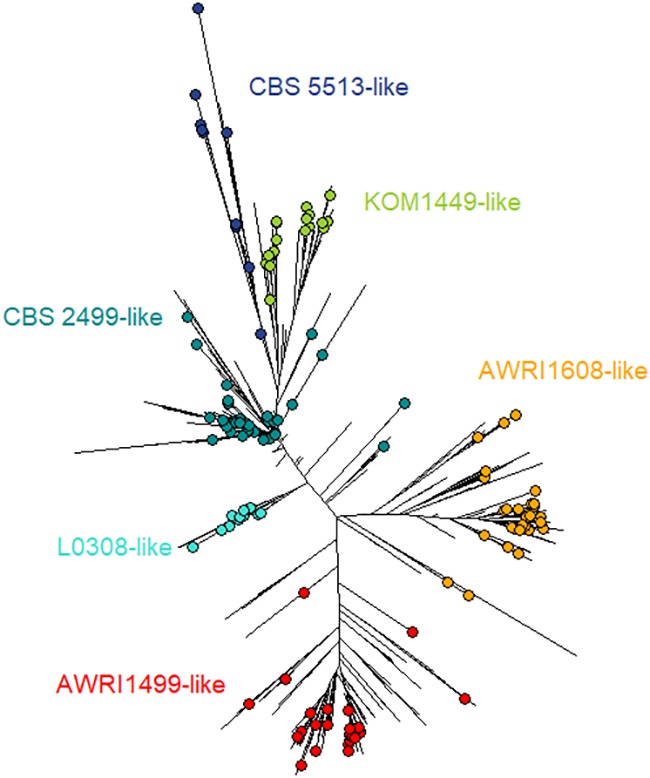
Dendrogram tree showing the 145 phenotyped *B. bruxellensis* isolates. The dendrogram tree includes 1488 isolates, and was built using 12 microsatellite markers, Bruvo’s distance, and NJ clustering, as described previously (Avramova et al., 2018). The 145 isolates used in this work are represented by colored circles. The six different colors correspond to the main genetic groups identified and were named from one isolate (e.g., L0308-like means genetic group close to L0308 strain).

In this study, 106 strains – in addition to the 39 strains tested previously (Avramova et al., 2018) – from different geographical and industrial fermentation origins were used based on their microsatellite profile (full protocol details and population dendrogram assessment in Avramova et al., 2018). Twelve microsatellite markers were used for genotyping, and a dendrogram was produced using Bruvo’s distance and Neighbor Joining (NJ) clustering. Those strains were evaluated for their tolerance to SO_2_ using the same protocol as previously described (Avramova et al., 2018) (details in the section “Sulfite Tolerance Assessment”) which made possible the combination of both datasets together to give a total of 145 strains (**Table 1** and **Figure 1**).

**Table 1:** Summary of the collection of 145 *Brettanomyces bruxellensis* strains used for sulfur dioxide tolerance assay.

Substrate	Beer (13); Cider (1); ethanol (2); Fruit wine (1); Kombucha (6); Tequila (6); Wine (107); NA (9)
Country	Argentina (1); Australia (9); Belgium (6); Brazil (4); Chile (3); Denmark (5); France (60); Germany (1); Italy (27); Mexico (6); Netherlands (1); New Zealand (1); Portugal (4); South Africa (6); Spain (2); Thailand (1); United Kingdom (1); Uruguay (1); United States (5); NA (1)
Vintage	1912 (1); 1926 (1); 1931 (1); 1938 (1); 1941 (1); 1949 (1); 1959 (1); 1990 (4); 1991 (2); 1992 (5); 1993 (1); 1994 (4); 1995 (2); 1998 (1); 2001 (6); 2002 (6); 2003 (6); 2003–2011 (2); 2004 (5); 2005 (2); 2006 (1); 2010 (1); 2011 (1); 2012 (17); 2013 (20); 2014 (19); 2015 (6); NA (27)
Genetic group	AWRI1499-like (32); AWRI1608-like (30); CBS 2499-like (42); CBS 5513-like (11); KOM1449-like (18); L0308-like (12)

*Full details available in **Supplementary Table S1**. NA stands for Not Available.*

### Sulfite Tolerance Assessment

The assay was performed in liquid medium containing 6.7 g.L^-1^ of YNB (Difco^TM^ Yeast Nitrogen Base, Becton, Dickinson and Company), 2.5 g.L^-1^ D-glucose, 2.5 g.L^-1^ D-Fructose, 5% (v/v) ethanol and increasing concentrations of potassium metabisulfite (PMB, K_2_S_2_O_5_, Thermo Fischer Scientific) in order to obtain 0, 0.2, 0.4, and 0.6 mg.L^-1^ mSO_2_ final concentrations. For the calculation of mSO_2_ it was considered that K_2_S_2_O_5_ corresponds to about 50% of total SO_2_ (therefore a solution of 10 g.L^-1^ K_2_S_2_O_5_ corresponds to approximately 5 g.L^-1^ total SO_2_). In order to deduce the final mSO_2_ concentration, the free SO_2_ concentration was assessed by aspiration/titration method. Then, the mSO_2_ was calculated by using the Henderson–Hasselbalch equation on dissociation constant p*K*1 (Divol et al., 2012). Ethanol concentration (5%) was chosen to allow growth of all strains, isolated from wine as well as from other fermentation niches with lower initial ethanol content. Final pH was adjusted to 3.5 (corresponding to an average value for pH generally encountered in red winemaking conditions) with phosphoric acid (1 M H_3_PO_4_) and the four media (corresponding to the four different concentrations of SO_2_) were filtered separately with 0.22 μm pore filter (Millipore).

Small-scale fermentations were performed in sterile 4 mL spectrophotometer cuvettes containing a sterile magnet stirrer (Dutscher, France). The cells were grown on YPD agar and inoculated into the YNB-based medium without SO_2_. After 96 h of pre-culture (the point at which all strains reached stationary phase), the cells were inoculated at OD_600nm_ 0.1 in a final volume of 3 mL. The inoculated medium was then covered with 300 μL of sterile silicone oil (Sigma-Aldrich) to avoid oxidation of the medium which could favor the free SO_2_ consumption. Then, the cuvette was capped with a plastic cap (Dutscher) and sealed with parafilm. A sterile needle was added by piercing the cap to allow CO_2_ release. These so-called nano-fermenters were then placed in a spectrophotometer cuvettes container box and on a 15 multi-positions magnetic stirrer plate at 25°C (the final temperature in the nano-fermenters was therefore 29°C due to the stirrer heating). Optical density (OD_600nm_) was measured every 24 h during at least 150 h to follow cell population growth until stationary phase was reached.

### Growth Parameter Calculation and Statistical Analyses

For each growth curve, the following three parameters were calculated: OD_max_ was the maximal OD reached at 600 nm and corresponded to the maximal population size, the lag phase (in hours) was the time between inoculation and the beginning of cell growth (5% maximal OD increase), and finally, the maximal growth rate was calculated (maximal number of division per hour based on the OD measurement divided by time).

Non-parametric Kruskal–Wallis tests were performed (α = 5%) to identify the means that were significantly different. All statistical analyses and graphs were produced using R language (R Development Core Team, 2010).

## Results

### Growth Behavior in Presence of SO_2_

**FIGURE 2:**
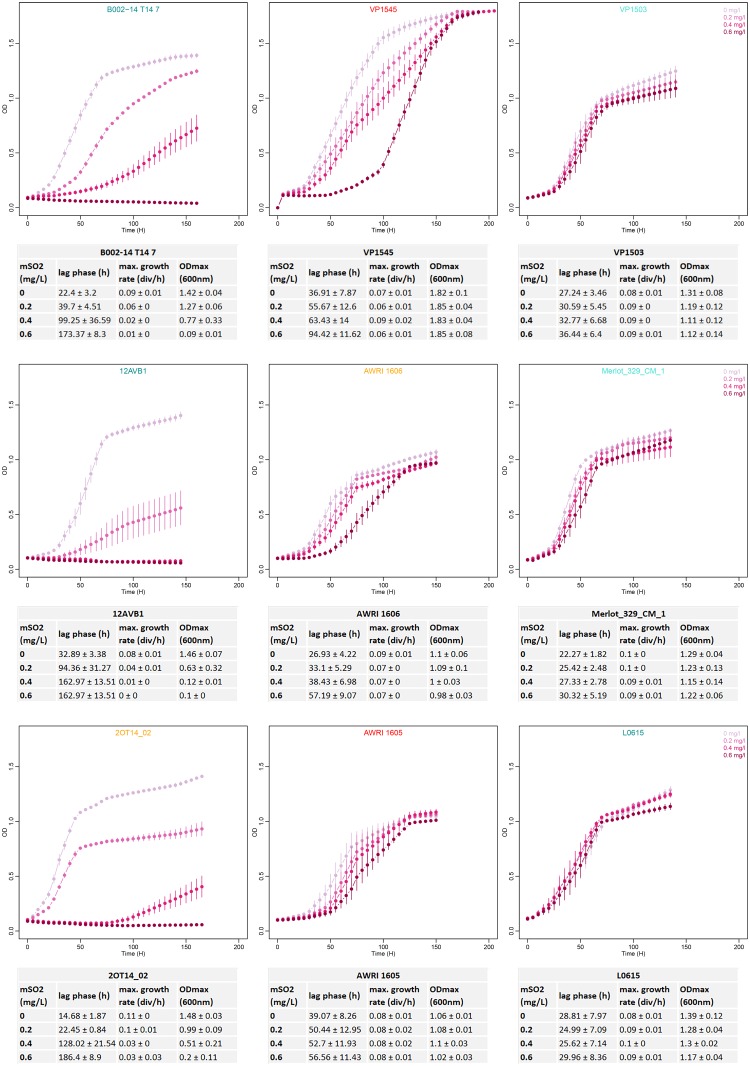
Examples of *B. bruxellensis* sensitive, tolerant, and resistant behavior at four mSO_2_ concentrations. Strains B002-14 T14 7, 12AVB1, and 2OT14_02 represent sensitive strains. VP1545, AWRI 1606, and AWRI 1605 are tolerant isolates and VP1503, Merlot_329_CM_1, and L0615 are examples of resistant strains. Each curve is built using the mean of three to four replicates, and error bars represent standard deviations and curve colors correspond to increasing SO_2_ concentration (light pink 0 mg/L mSO_2_ to dark pink 0.6 mg/L mSO_2_). The estimated growth parameters (lag phase, maximal growth rate, and maximal OD) are shown below each curve, with mean ± standard deviation.

The growth behavior of 145 strains of *B. bruxellensis* was evaluated regarding sensitivity to sulfite treatment. The selected strains were distributed amongst the six main genetic groups defined using microsatellite markers and were representative of the genetic diversity of the species (**Figure 1**): CBS 2499-like, KOM1449-like, AWRI1608-like, AWRI1499-like, CBS 5513-like, and L0308-like groups were represented by 42, 18, 30, 32, 11, and 12 strains, respectively (**Table 2**). A total of >2050 small-scale fermentations were performed, corresponding to each strain tested at increasing concentrations of mSO_2_ (0, 0.2, 0.4, and 0.6 mg.L^-1^) at least in triplicate. The strains had different response to sulfur dioxide concentrations in means of lag phase, maximal growth rate, and maximum OD. Depending on the growth parameters’ variation (**Supplementary Table S1**), three growth behaviors were defined (**Figure 2**). Sensitive strains showed significantly longer lag phase and slower growth rate and/or lower maximum OD in presence of increasing concentrations of SO_2_: for example, strain B002-14 T14 7 (**Figure 2**) showed 22.4, 39.7, 99.2, and 173.4 h of lag phase with 0, 0.2, 0.4, and 0.6 mg.L^-1^ mSO_2_, respectively. Maximal growth rate decreased along sulfite concentration with 0.09, 0.06, 0.02, and 0.01 division/h, and OD_max_ decreased drastically with 1.42, 1.27, 0.77, and 0.09 OD. The same pattern (increased lag-phase, decreased growth rate, and decreased OD_max_) was observed for strains 12AVB1 and 2OT14_02 (**Figure 2**). The degree of sensitivity varied depending on the isolates: some strains showed low growth in presence of 0.2 mg.L^-1^ mSO_2_ like strain CBS 3025 which OD_max_ drops from 1.92 to 0.13 at 0 and 0.2 mg.L^-1^ mSO_2_, respectively, or strain 12AVB1 that shows a twofold decrease of OD_max_ between 0 and 0.2 mg.L^-1^ mSO_2_ (1.46 to 0.63, see **Supplementary Table S1**). Other isolates showed close to normal growth at 0.2 mg.L^-1^ mSO_2_ (OD_max_ > 1), but low/no growth at 0.4 mg.L^-1^ mSO_2_ (AWRI1615, L02/E2 AZ, L14160, L14186, YJS5447, etc.). Finally, other strains, although showing a significant growth decrease, were still able to show moderate growth at 0.6 mg.L^-1^ mSO_2_: for example, lag-phase of UWOPS 92–297.4 was drastically impacted, from 7 and 10 h (0 and 0.2 mg.L^-1^ mSO_2_) to 154 and 171 h (0.4 and 0.6 mg.L^-1^ mSO_2_). Its OD_max_ was also clearly impacted, ranging from 1.29 to 0.54 (at 0 and 0.6 mg.L^-1^ mSO_2_, respectively), yet with a residual growth. In conclusion, all strains considered to be sensitive had significantly longer lag phase and slower growth rate and/or lower maximum OD in presence of increasing concentrations of SO_2_. However, the sulfite concentration at which growth began to be impacted varied, as well as the level of growth’s decrease.

**Table 2:** Number of isolates by genetic group and phenotype.

Genetic group	Sensitive	Tolerant	Resistant	Total
CBS 2499-like	38	1	3	42
KOM1449-like	14	3	1	18
AWRI1608-like	27	2	1	30
AWRI1499-like	4	7	21	32
CBS 5513-like	10	0	1	11
L0308-like	0	1	11	12
Total	93	14	38	145

By contrast, tolerant strains displayed increased lag phase with SO_2_ increase, while others growth parameters (maximal growth rate and maximal OD) remained statistically unchanged (Kruskal–Wallis test, α = 0.05). For example, strain VP1545 (**Figure 2**) showed varying lag phase (36.9, 55.7, 63.4, and 94.4 h at 0, 0.2, 0.4, and 0.6 mg.L^-1^ mSO_2_, respectively), but unchanged maximal growth rate (0.07–0.09 division/h) and OD_max_ (1.82–1.85 OD). The same pattern is observed for AWRI 1606 (lag-phase ranging from 27 to 57 h) or AWRI 1605 (lag-phase between 39 and 57 h). Finally, strains for which none parameters were significantly impacted whatever the SO_2_ concentrations were considered as resistant: VP1503 (**Figure 2**) had unchanged lag phase of 27.2 to 36.4 h, maximal growth rate of 0.08–0.09 division/h and OD_max_ of 1.11–1.31 OD. Identically, Merlot_329_M_1 and L0615 showed identical growth’s kinetics whatever the SO_2_ concentrations tested.

### Relationship Between SO_2_ Sensitivity and Genetic Groups

**FIGURE 3:**
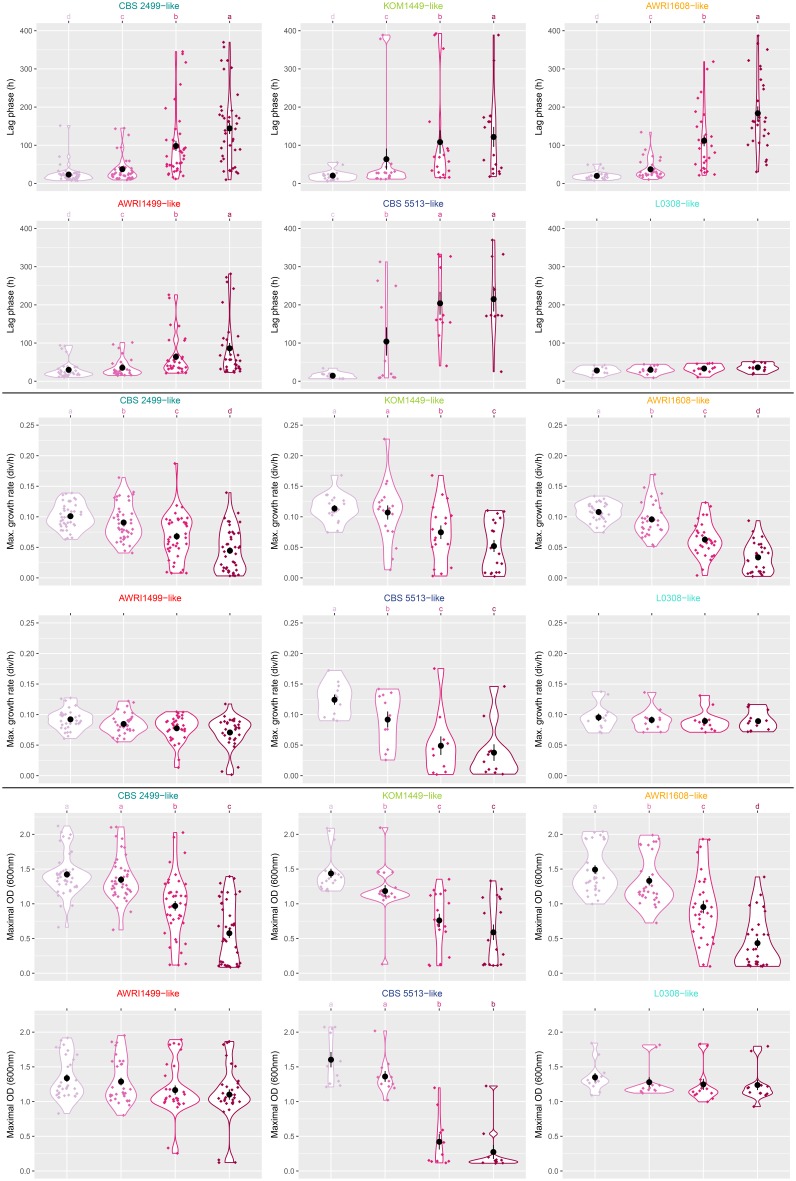
Violin plots for three growth parameters and six genetic groups of *B. bruxellensis*. Three growth parameters were represented: lag phase (h), maximum growth rate (division per hour), and maximum OD (600 nm). For each genetic group, numeric values (corresponding to the different strains) are represented as diamonds, the corresponding probability densities are represented as plain traits, means, and standard errors are represented by black circles and segments, respectively. Increasing SO_2_ concentrations are represented by the same coloring (pink shades, light pink corresponding to 0 mg/L and darker color representing increasing SO_2_ concentrations) as in **Figure 2**. The plots were obtained using *ggplot2* package (R). Top letters represent significance groups as defined by Kruskal–Wallis test (*agricolae* package, *p*-value < 0.05). Absence of top letters indicates non-significantly different sulfur conditions.

When analyzed globally, clear differences between the different genetic groups were observed (**Figure 3**): the L0308-like group showed mostly resistant behavior (invariant growth parameters whatever sulfite concentration). The AWRI1499-like group showed mostly unchanged maximal growth rate and OD, and showed either unchanged lag phase (resistant strains) or poorly increased lag phase (tolerant strains). All other groups were mostly sensitive to sulfite treatments, with an important variability amongst strains regarding to their degree of sensitivity.

A more precise analysis, strain by strain, was performed (**Table 2** and **Supplementary Figure S1**). An important proportion of the tested isolates (52/145, 36%) were either tolerant or resistant to sulfite treatments, and this was strongly related to genetic groups. For example, all 12 isolates of the L0308-like group were either resistant (11) or tolerant (1) to sulfite treatments. Similarly, amongst the 32 isolates tested for the AWRI1499-like group, 21 were resistant, 7 tolerant, and only 4 sensitive to sulfite treatments. This confirms that, globally, most isolates from L0308-like and AWRI1499-like groups are resistant/tolerant to sulfite. By contrast, the other groups contained mostly sensitive strains (38/42 for CBS 2499-like; 14/18 for KOM1449-like; 27/30 for AWRI1608-like; 10/11 for CBS 5513-like).

In addition, 46 out of 52 tolerant or resistant strains were isolated from wine (**Supplementary Table S1**). Indeed, the proportion of tolerant/resistant isolates from wine represented 43% (46/107).

## Discussion

Sulfur dioxide is usually used by winemakers as preventive or curative treatment for spoilage microorganisms including *B. bruxellensis* contamination. Concentrations of 0.2 to 0.5 mg.L^-1^ molecular SO_2_ are typically reported to inhibit growth in wine (Conterno et al., 2006; Barata et al., 2008). However, some *B. bruxellensis* strains were shown to be rather sulfite tolerant (Barata et al., 2008; Vigentini et al., 2008; Curtin et al., 2012; Agnolucci et al., 2014; Avramova et al., 2018) and sulfite efficiency was elucidated as population level dependent (Longin et al., 2016). Previous studies highlighted genotype-dependent tolerance to sulfur dioxide for *B. bruxellensis* among Australian isolates with AFLP markers (Curtin et al., 2012), and this was recently confirmed for 39 isolates analyzed with microsatellite markers (Avramova et al., 2018). Taking into account the high intra-species genetic diversity of *B. bruxellensis*, 106 additional isolates from various origins were included to the previous phenotypic test to confirm the link between genotype and SO_2_ tolerance at larger and finer scale. Here, we show that 36% of *B. bruxellensis* isolates are resistant/tolerant to sulfite (up to 43% amongst wine isolates), and we confirm the relationship between genetic groups and survival patterns in presence of sulfite treatments.

In our previous study, it was noticed that representatives of the L0308-like group exhibited a peculiar profile characterized by unmodified growth parameters at all tested SO_2_ concentrations. However, these observations were based on only two isolates with similar origin (Avramova et al., 2018). To complete these results, we analyzed 9 additional L0308-like strains from different origins and confirmed their (mostly) resistant phenotype. Here, a resistant phenotype corresponds to behavior for which there were no significant differences for all studied growth parameters at increasing SO_2_ concentration. On the other hand, tolerant strains were those for which lag phase was modified with SO_2_ increase. Those two terms are used in clinical microbiology, where they serve to describe microbial pathogenicity (Anderson, 2005; Brauner et al., 2016). Often, tolerance is related to the capacity of the organism to survive under inhibition by an agent, whereas resistance is linked to the capacity to actively proliferate in presence of antibiotic, and is measured as minimum inhibitory concentration or fitness (Anderson, 2005). The peculiarity of SO_2_ application, however, is that the main active antimicrobial fraction (mSO_2_) of this agent depends on environmental parameters (such as temperature, alcohol content, and mainly pH) and that the active fraction decreases over time due to free SO_2_ combination. Furthermore, *B. bruxellensis* is able to enter a VBNC (viable but not cultivable) state after sulfites addition (du Toit et al., 2005; Agnolucci et al., 2010; Serpaggi et al., 2012; Capozzi et al., 2016; Longin et al., 2016), followed by growth recovery when sulfites decrease over time. In winemaking, sulfite levels are regularly re-adjusted at different time intervals, thus creating seasonality in SO_2_ administration during the winemaking process. In these conditions, the actual survival of *B. bruxellensis* in wine could be related to (i) survival and growth besides initial “hit” with SO_2_, that could be related to resistant-type mechanism and (ii) survival at the initial SO_2_ “hit” and until a stage when mSO_2_ concentration is lower in the medium, followed by growth recovery that could be described as tolerance mechanism. Indeed, resistant and tolerant phenotypes are often interconnected and related to different types of metabolism and cell structure differences. In clinical microbiology, it is suggested that tolerant and resistant strains should be treated differently: resistant should be treated with higher doses and shorter treatment, whereas tolerant strains should be treated with lower doses but extended treatment duration (Brauner et al., 2016). The detection of both resistant and tolerant growth profiles in the present dataset suggests that *B. bruxellensis* strains have developed not one, but multiple strategies to cope with SO_2_ present in wine.

Here, the majority of tolerant or resistant strains were isolated from wine (46 out of 52). This suggests a strong link between SO_2_ exposure related to the winemaking industry and *B. bruxellensis* survival in presence of SO_2_ (Curtin et al., 2012). This data highlights the role of SO_2_, and therefore human activity, in shaping *B. bruxellensis* population structure, which was also suggested in previous studies (Curtin et al., 2012; Avramova et al., 2018). Sulfur dioxide resistance is broadly studied in *S. cerevisiae* and the main molecular mechanisms explaining this phenotype is efflux through Ssu1p active pump (Park and Bakalinsky, 2000; Perez-Ortin et al., 2002; Nardi et al., 2010). It was demonstrated that SSU1-R allele, which is involved in SO_2_ resistance, is the product of reciprocal translocation between chromosomes VII and XVI, thus highlighting the importance of gross chromosomal rearrangements in the adaptive evolution of *S. cerevisiae* (Perez-Ortin et al., 2002). Later, another translocation involved in SO_2_ tolerance (XV-t-XVI) was shown to shorten lag phase in presence of SO_2_, thus conferring relative selective advantage compared to non-translocated XVI strains (Zimmer et al., 2014). Following those studies, it was suggested that those translocations were empirically selected by humans (Perez-Ortin et al., 2002; Zimmer et al., 2014). The lack of effect of SO_2_ on lag phase observed for the resistant *B. bruxellensis* strains could be related to similar mechanisms. Indeed, allele specific expression of efflux pump BbSSU1 was detected by comparative transcriptomics (Curtin et al., 2015). However, the molecular mechanisms underlying resistant phenotype in *B. bruxellensis* remain to be elucidated. As for the tolerant strains, the longer lag phase would reflect the time needed for the adaptation through complex mechanisms or the survival until a lower mSO_2_ concentration is attained in the medium. Using staining with propidium iodide detection by flow cytometer analysis, Longin et al. (2016) showed that sulfite induces increased yeast cell permeability, which probably leads to cell death. The ability of cells to restore functional cell permeability could constitute another sulfite adaptation mechanism for *B. bruxellensis*. The SO_2_ molecule has various effects on the cell structure, metabolism, and genome (Divol et al., 2012), and the corresponding mechanisms could include synthesis of binding molecules (like acetaldehyde), specific membrane structure, etc (Divol et al., 2012).

The sensitivity/survival phenotype in presence of SO_2_ correlates with genotypic profiles defined by microsatellite analysis in a set of 145 representative strains (Avramova et al., 2018). The groups CBS 2499-like, KOM1449-like, AWRI1608-like, and CBS 5513-like are all susceptible to SO_2_ presence in synthetic medium. On the contrary, AWRI1499-like and L0308-like survived in presence of high concentrations of mSO_2_. This behavior was confirmed by independent study (Longin et al., 2016) performed in wine medium, where the strain L0417 (AWRI1499-like) was demonstrated to be more tolerant than L02E2 (CBS 2499-like). The use of microsatellites as selection markers was previously proposed for *S. cerevisiae* wine strains (Franco-Duarte et al., 2009, 2014). In the latter work, 30 different phenotypes were analyzed, and SO_2_ tolerance was one of the factors that correlate the most with microsatellite patterns. In the winemaking context, SO_2_ tolerance is a positive trait for the selection of *S. cerevisiae*, whereas it is the opposite for *B. bruxellensis* strains, for which it is directly related to spoilage potential. Defining SO_2_ tolerance through genetic markers can therefore be used as an efficient tool to adapt antimicrobial treatment in winery. Similar methods are used for resistance prediction for pathogenic fungi (Park and Perlin, 2005; Irinyi et al., 2015). Namely, in the case of *C. albicans*, PCR-based methods were proposed for the detection of mutations related to fluconazole resistance (Park and Perlin, 2005). This method allows the adoption of alternative techniques to cope with this microorganism. Contrary to fluconazole, SO_2_ has a very broad range of actions on the cell at structural, genetic, and metabolic level (White et al., 2002; Divol et al., 2012), and detection method of specific mutation responsible for resistance would be a challenge. Therefore, the strong correlation between genotype and SO_2_ tolerance presents a reliable alternative for the prediction of this phenotype through microsatellite analysis. Indeed, resistant/tolerant genotypes can be reliably predicted: 91% (40/44 strains) of the AWRI1499-like and L0308-like isolates are actually tolerant or resistant to sulfite. For comparison, this percentage was 91% for *C. albicans* (based on 32 isolates) when using targeted PCR (Park and Perlin, 2005). Combined with the fact that clonal populations of *B. bruxellensis* strains were isolated over a long period of time in the same winery (Albertin et al., 2014), the use of microsatellite markers is also applicable as a prediction method based on spoilage populations from previous vintages. Hence, the use of microsatellite markers is a reliable method for predicting spoilage potential in means of SO_2_ tolerance for *B. bruxellensis* populations, although a bit expensive and time-consuming for routine analysis. Therefore, we developed an alternative analysis, based on a single duplex PCR and classical gel electrophoresis migration that indicates (i) whether the isolates belong to *B. bruxellensis* species and (ii) their sulfur dioxide sensitivity (Albertin et al., 2017a, 2018). This approach was patented (Albertin et al., 2017b) and is compatible with day-to-day analysis by oenological laboratories. Such diagnosis could allow application of adequate antimicrobial techniques according to the survival mechanism in presence of SO_2_ of the contaminating *B. bruxellensis* population, and thus to assure efficient spoilage prevention with minimal intervention.

## Author Contributions

IM-P and WA conceived the study. MA, AV-C, and JM performed the experiments. All authors analyzed the data and wrote the manuscript.

## Conflict of Interest Statement

The authors declare that the research was conducted in the absence of any commercial or financial relationships that could be construed as a potential conflict of interest.

## Supplementary Material

TABLE S1 Growth parameters of *B. bruxellensis* strains in different concentrations of sulphur dioxide. ^a^AWRI, The Australian Wine Research Institute, Glen Osmond, SA, Australia; CBS, Centraalbureau voor Schimmelcultures, Fungal Biodiversity Centre, Utrecht, Netherlands; CRBO, Centre de Ressources Biologiques Œnologie, Villenave d’Ornon, France; HGU, Hochschule Geisenheim University, Geisenheim, Germany; ICV, Institut coopératif du vin, Lattes, France; Inter-Rhone, Inter Rhône, Avignon, France; ISA, Instituto Superior de Agronomia, Lisbon, Portugal; ISVV, Institut des Sciences de la Vigne et du Vin, Villenave d’Ornon, France; IUVV, Institut Universitaire de la Vigne et du Vin Jules Guyot, Dijon, France; Microflora, Microflora, Villenave d’Ornon, France; UFPE, Federal University of Pernambuco, Recife, Brazil; UNIB, Université de Brest, Brest, France; UNIFG, University of Foggia, Foggia, Italy; UNINA, University of Naples Federico II, Napoli, Italy; UNISTRA, University of Strasbourg, Strasbourg, France; UWOPS, Culture collection of the University of Western Ontario, London, On, Canada; NA, Not Available.

FIGURE S1 Growth parameters of 145 *B. bruxellensis* isolates grown at different SO_2_ concentrations. Growth parameters lag phase (h), maximum growth rate (division per hour), and maximum OD (600 nm) are presented for 145 isolates. Isolates are clustered by genetic group as defined previously (Avramova et al., 2018), in order: CBS 2499-like group (dark cyan), KOM1449-like (light green), AWRI1608-like (orange), AWRI1499-like (red), dark blue (CBS 5513-like), turquoise (L0308-like). Vertical traits present standard deviations.

## References

[B1] Agnolucci M. Cristani C. Maggini S. Rea F. Cossu A. Tirelli A. (2014). Impact of sulphur dioxide on the viability, culturability, and volatile phenol production of *Dekkera bruxellensis* in wine. *Ann. Microbiol.* 64 653–659. 10.1007/s13213-013-0698-6

[B2] Agnolucci M. Rea F. Sbrana C. Cristani C. Fracassetti D. Tirelli A. (2010). Sulphur dioxide affects culturability and volatile phenol production by *Brettanomyces*/*Dekkera bruxellensis*. *Int. J. Food Microbiol.* 143 76–80. 10.1016/j.ijfoodmicro.2010.07.022 20705352

[B3] Albertin W. Avramova M. Cibrario A. Ballestra P. Dols-Lafargue M. Curtin C. (2017a). *Brettanomyces bruxellensis*: diversité génétique et sensibilité aux sulfites. *Rev. Oenol. Tech. Vitivinicoles Oenol.* 44 31–33.

[B4] Albertin W. Masneuf-Pomarede I. Peltier E. (2017b). Method for Analysing a Sample to Detect the Presence of Sulphite-Resistant Yeasts of the *Brettanomyces bruxellensis* Species and Kit for Implementing Same. France patent no. PCT/FR2016/052701.

[B5] Albertin W. Avramova M. Maupeu J. Vallet-Courbin A. Cibrario A. Dols-Lafargue M. (2018). Pratique du sulfitage. *Brettanomyces bruxellensis* s’adapte ! *Union Girondine Vins Bord.* 1153 46–48.

[B6] Albertin W. Panfili A. Miot-Sertier C. Goulielmakis A. Delcamp A. Salin F. (2014). Development of microsatellite markers for the rapid and reliable genotyping of *Brettanomyces bruxellensis* at strain level. *Food Microbiol.* 42 188–195. 10.1016/j.fm.2014.03.012 24929736

[B7] Anderson J. B. (2005). Evolution of antifungal-drug resistance: mechanisms and pathogen fitness. *Nat. Rev. Microbiol.* 3 547–556. 10.1038/nrmicro1179 15953931

[B8] Avramova M. Cibrario A. Peltier E. Coton M. Coton E. Schacherer J. (2018). *Brettanomyces bruxellensis* population survey reveals a diploid-triploid complex structured according to substrate of isolation and geographical distribution. *Sci. Rep.* 8:4136. 10.1038/s41598-018-22580-7 29515178 PMC5841430

[B9] Barata A. Caldeira J. Botelheiro R. Pagliara D. Malfeito-Ferreira M. Loureiro V. (2008). Survival patterns of *Dekkera bruxellensis* in wines and inhibitory effect of sulphur dioxide. *Int. J. Food Microbiol.* 121 201–207. 10.1016/j.ijfoodmicro.2007.11.020 18077036

[B10] Berbegal C. Garofalo C. Russo P. Pati S. Capozzi V. Spano G. (2017). Use of autochthonous yeasts and bacteria in order to control *Brettanomyces bruxellensis* in wine. *Fermentation* 3:65 10.3390/fermentation3040065

[B11] Berbegal C. Spano G. Fragasso M. Grieco F. Russo P. Capozzi V. (2018). Starter cultures as biocontrol strategy to prevent *Brettanomyces bruxellensis* proliferation in wine. *Appl. Microbiol. Biotechnol.* 102 569–576. 10.1007/s00253-017-8666-x 29189899 PMC5756568

[B12] Brauner A. Fridman O. Gefen O. Balaban N. Q. (2016). Distinguishing between resistance, tolerance and persistence to antibiotic treatment. *Nat. Rev. Microbiol.* 14 320–330. 10.1038/nrmicro.2016.34 27080241

[B13] Capozzi V. Di Toro M. R. Grieco F. Michelotti V. Salma M. Lamontanara A. (2016). Viable But Not Culturable (VBNC) state of *Brettanomyces bruxellensis* in wine: new insights on molecular basis of VBNC behaviour using a transcriptomic approach. *Food Microbiol.* 59 196–204. 10.1016/j.fm.2016.06.007 27375260

[B14] Chatonnet P. Dubourdieu D. Boidron J.-N. Pons M. (1992). The origin of ethylphenols in wines. *J. Sci. Food Agric.* 60 165–178. 10.1002/jsfa.2740600205 18576949

[B15] Conterno L. Joseph C. M. L. Arvik T. J. Henick-Kling T. Bisson L. F. (2006). Genetic and physiological characterization of *Brettanomyces bruxellensis* strains isolated from wines. *Am. J. Enol. Vitic.* 57 139–147.

[B16] Curtin C. Joseph L. Zeppel R. Albertin W. Masneuf-Pomarede I. Bisson L. F. (2015). “Genomic and transcriptomic landscape of the industrial yeast species *Brettanomyces bruxellensis*,” in *The 32nd International Specialized Symposium on Yeasts (ISSY32)*, Perugia [accessed September 13–17, 2015].

[B17] Curtin C. Kennedy E. Henschke P. A. (2012). Genotype-dependent sulphite tolerance of Australian *Dekkera* (Brettanomyces) *bruxellensis* wine isolates. *Lett. Appl. Microbiol.* 55 56–61. 10.1111/j.1472-765X.2012.03257.x 22537453

[B18] Delfini C. Gaia P. Schellino R. Strano M. Pagliara A. Ambrò S. (2002). Fermentability of grape must after inhibition with dimethyl dicarbonate (DMDC). *J. Agric. Food Chem.* 50 5605–5611. 10.1021/jf0256337 12236685

[B19] Divol B. Toit M. Duckitt E. (2012). Surviving in the presence of sulphur dioxide: strategies developed by wine yeasts. *Appl. Microbiol. Biotechnol.* 95 601–613. 10.1007/s00253-012-4186-x 22669635

[B20] du Toit W. J. Pretorius I. S. Lonvaud-Funel A. (2005). The effect of sulphur dioxide and oxygen on the viability and culturability of a strain of *Acetobacter pasteurianus* and a strain of *Brettanomyces bruxellensis* isolated from wine. *J. Appl. Microbiol.* 98 862–871. 10.1111/j.1365-2672.2004.02549.x 15752332

[B21] Fabrizio V. Vigentini I. Parisi N. Picozzi C. Compagno C. Foschino R. (2015). Heat inactivation of wine spoilage yeast *Dekkera bruxellensis* by hot water treatment. *Lett. Appl. Microbiol.* 61 186–191. 10.1111/lam.12444 25989358

[B22] Fleet G. H. (2003). Yeast interactions and wine flavour. *Int. J. Food Microbiol.* 86 11–22. 10.1016/S0168-1605(03)00245-9 12892919

[B23] Francesca C. Maurizio C. (2011). *Kluyveromyces wickerhamii* killer toxin: purification and activity towards *Brettanomyces*/*Dekkera* yeasts in grape must. *FEMS Microbiol. Lett.* 316 77–82. 10.1111/j.1574-6968.2010.02194.x 21204930

[B24] Franco-Duarte R. Mendes I. Umek L. Drumonde-Neves J. Zupan B. Schuller D. (2014). Computational models reveal genotype-phenotype associations in *Saccharomyces cerevisiae*. *Yeast* 31 265–277. 10.1002/yea.3016 24752995

[B25] Franco-Duarte R. Umek L. Zupan B. Schuller D. (2009). Computational approaches for the genetic and phenotypic characterization of a *Saccharomyces cerevisiae* wine yeast collection. *Yeast* 26 675–692. 10.1002/yea.1728 19894212

[B26] González-Arenzana L. López-Alfaro I. Garde-Cerdán T. Portu J. López R. Santamaría P. (2018). Microbial inactivation and MLF performances of Tempranillo Rioja wines treated with PEF after alcoholic fermentation. *Int. J. Food Microbiol.* 269 19–26. 10.1016/j.ijfoodmicro.2018.01.008 29358132

[B27] González-Arenzana L. Sevenich R. Rauh C. López R. Knorr D. López-Alfaro I. (2016). Inactivation of *Brettanomyces bruxellensis* by high hydrostatic pressure technology. *Food Control* 59 188–195. 10.1016/j.foodcont.2015.04.038

[B28] Heresztyn T. (1986). Metabolism of volatile phenolic compounds from hydroxycinnamic acids by *Brettanomyces* yeast. *Arch. Microbiol.* 146 96–98. 10.1007/bf00690165

[B29] Irinyi L. Serena C. Garcia-Hermoso D. Arabatzis M. Desnos-Ollivier M. Vu D. (2015). International society of human and animal mycology (ISHAM)-ITS reference DNA barcoding database–the quality controlled standard tool for routine identification of human and animal pathogenic fungi. *Med. Mycol.* 53 313–337. 10.1093/mmy/myv008 25802363

[B30] Longin C. Degueurce C. Julliat F. Guilloux-Benatier M. Rousseaux S. Alexandre H. (2016). Efficiency of population-dependent sulfite against *Brettanomyces bruxellensis* in red wine. *Food Res. Int.* 89(Pt 1), 620–630. 10.1016/j.foodres.2016.09.019 28460958

[B31] Luo H. Schmid F. Grbin P. R. Jiranek V. (2012). Viability of common wine spoilage organisms after exposure to high power ultrasonics. *Ultrason. Sonochem.* 19 415–420. 10.1016/j.ultsonch.2011.06.009 21978847

[B32] Lustrato G. Vigentini I. De Leonardis A. Alfano G. Tirelli A. Foschino R. (2010). Inactivation of wine spoilage yeasts *Dekkera bruxellensis* using low electric current treatment (LEC). *J. Appl. Microbiol.* 109 594–604. 10.1111/j.1365-2672.2010.04686.x 20148995

[B33] Mehlomakulu N. N. Setati M. E. Divol B. (2014). Characterization of novel killer toxins secreted by wine-related non-Saccharomyces yeasts and their action on Brettanomyces spp. *Int. J. Food Microbiol.* 188 83–91. 10.1016/j.ijfoodmicro.2014.07.015 25087208

[B34] Nardi T. Corich V. Giacomini A. Blondin B. (2010). A sulphite-inducible form of the sulphite efflux gene *SSU1* in a *Saccharomyces cerevisiae* wine yeast. *Microbiology* 156(Pt 6), 1686–1696. 10.1099/mic.0.036723-0 20203053

[B35] Park H. Bakalinsky A. T. (2000). SSU1 mediates sulphite efflux in *Saccharomyces cerevisiae*. *Yeast* 16 881–888. 10.1002/1097-0061(200007)16:10<881::AID-YEA576>3.0.CO;2-3 10870099

[B36] Park S. Perlin D. S. (2005). Establishing surrogate markers for fluconazole resistance in *Candida albicans*. *Microb. Drug Resist.* 11 232–238. 10.1089/mdr.2005.11.232 16201925

[B37] Perez-Ortin J. E. Querol A. Puig S. Barrio E. (2002). Molecular characterization of a chromosomal rearrangement involved in the adaptive evolution of yeast strains. *Genome Res.* 12 1533–1539. 10.1101/gr.436602 12368245 PMC187534

[B38] Petrova B. Cartwright Z. M. Edwards C. G. (2016). Effectiveness of chitosan preparations against *Brettanomyces bruxellensis* grown in culture media and red wines. *OENO One* 50 49–56. 10.20870/oeno-one.2016.50.1.54

[B39] R Development Core Team (2010). *R: A Language and Environment for Statistical Computing*. Vienna: R Foundation for Statistical Computing.

[B40] Ribéreau-Gayon P. Dubourdieu D. Donèche B. Lonvaud A. (2006). *Handbook of Enology: The Microbiology of Wine and Vinifications*. Hoboken, NJ: John Wiley & Sons, Ltd. 10.1002/0470010398

[B41] Serpaggi V. Remize F. Recorbet G. Gaudot-Dumas E. Sequeira-Le Grand A. Alexandre H. (2012). Characterization of the “viable but nonculturable” (VBNC) state in the wine spoilage yeast Brettanomyces. *Food Microbiol.* 30 438–447. 10.1016/j.fm.2011.12.020 22365358

[B42] Taillandier P. Joannis-Cassan C. Jentzer J. B. Gautier S. Sieczkowski N. Granes D. (2015). Effect of a fungal chitosan preparation on *Brettanomyces bruxellensis*, a wine contaminant. *J. Appl. Microbiol.* 118 123–131. 10.1111/jam.12682 25363885

[B43] Umiker N. L. Descenzo R. A. Lee J. Edwards C. G. (2013). Removal of *Brettanomyces bruxellensis* from red wine using membrane filtration. *J. Food Process. Preserv.* 37 799–805. 10.1111/j.1745-4549.2012.00702.x

[B44] Vigentini I. Romano A. Compagno C. Merico A. Molinari F. Tirelli A. (2008). Physiological and oenological traits of different Dekkera/*Brettanomyces bruxellensis* strains under wine-model conditions. *FEMS Yeast Res.* 8 1087–1096. 10.1111/j.1567-1364.2008.00395.x 18565109

[B45] White T. C. Holleman S. Dy F. Mirels L. F. Stevens D. A. (2002). Resistance mechanisms in clinical isolates of *Candida albicans*. *Antimicrob. Agents Chemother.* 46 1704–1713. 10.1128/AAC.46.6.1704-1713.2002 12019079 PMC127245

[B46] Zimmer A. Durand C. Loira N. Durrens P. Sherman D. J. Marullo P. (2014). QTL dissection of lag phase in wine fermentation reveals a new translocation responsible for *Saccharomyces cerevisiae* adaptation to sulfite. *PLoS One* 9:e86298. 10.1371/journal.pone.0086298 24489712 PMC3904918

