# Supplementary figures and images for: Molecular Diagnosis of Brettanomyces bruxellensis’ Sulfur Dioxide Sensitivity Through Genotype Specific Method
Source: Front Microbiol. 2018 Jun 11;9:1260. doi: 10.3389/fmicb.2018.01260 (PMC6004410; doi:10.3389/fmicb.2018.01260)

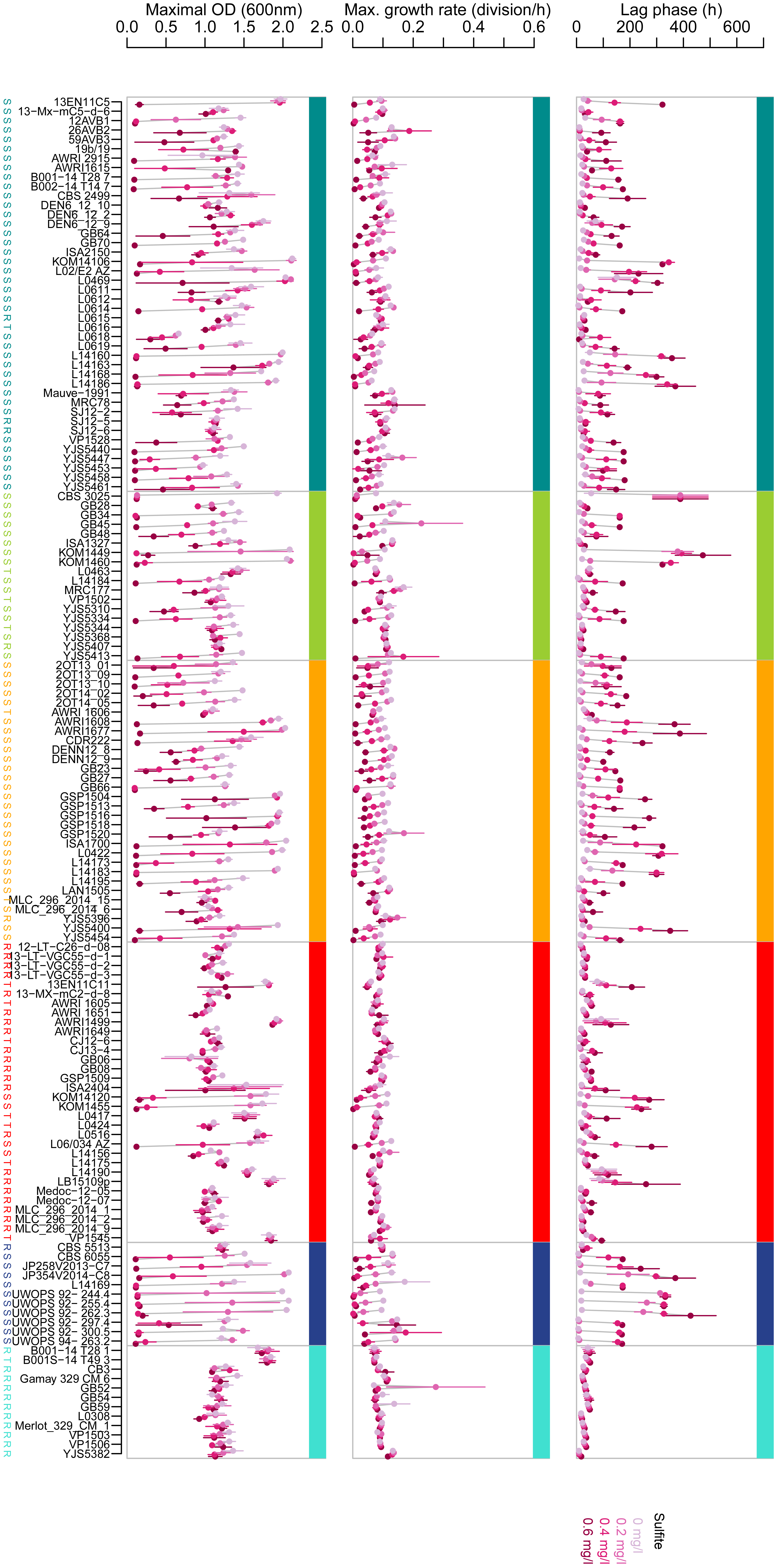

Supplement: FIGURE S1 — Growth parameters of 145 B. bruxellensis isolates grown at different SO2 concentrations. Growth parameters lag phase (h), maximum growth rate (division per hour), and maximum OD (600 nm) are presented for 145 isolates. Isolates are clustered by genetic group as defined previously (Avramova et al., 2018), in order: CBS 2499-like group (dark cyan), KOM1449-like (light green), AWRI1608-like (orange), AWRI1499-like (red), dark blue (CBS 5513-like), turquoise (L0308-like). Vertical traits present standard deviations. [file Image_1.TIF]
